# Refining Operational Practice for Controlling Introduced European Rabbits on Agricultural Lands in New Zealand

**DOI:** 10.1371/journal.pone.0158078

**Published:** 2016-06-24

**Authors:** A. David M. Latham, M. Cecilia Latham, Graham Nugent, James Smith, Bruce Warburton

**Affiliations:** Wildlife Ecology & Management, Landcare Research, P.O. Box 69040, Lincoln 7640, New Zealand; University of Southern Queensland, AUSTRALIA

## Abstract

European rabbits (*Oryctolagus cuniculus*) pose a major threat to agricultural production and conservation values in several countries. In New Zealand, population control via poisoning is a frontline method for limiting rabbit damage, with large areas commonly treated using the metabolic toxin sodium fluoroacetate (‘1080’) delivered in bait via aerial dispersal. However, this method is expensive and the high application rates of the active ingredient cause public antipathy towards it. To guide reductions in cost and toxin usage, we evaluated the economics and efficacy of rabbit control using an experimental approach of sowing 1080-bait in strips instead of the commonly-used broadcast sowing method (i.e. complete coverage). Over a 4-year period we studied aerial delivery of 0.02% 1080 on diced carrot bait over ~3500 ha of rabbit-prone land in the North and South islands. In each case, experimental sowing via strip patterns using 10–15 kg of bait per hectare was compared with the current best practice of aerial broadcast sowing at 30–35 kg/ha. Operational kill rates exceeded 87% in all but one case and averaged 93–94% across a total of 19 treatment replicates under comparable conditions; there was no statistical difference in overall efficacy observed between the two sowing methods. We project that strip-sowing could reduce by two thirds the amount of active 1080 applied per hectare in aerial control operations against rabbits, both reducing the non-target poisoning risk and promoting cost savings to farming operations. These results indicate that, similarly to the recently-highlighted benefits of adopting strip-sowing for poison control of introduced brushtail possums (*Trichosurus vulpecula*) in New Zealand, aerial strip-sowing of toxic bait could also be considered a best practice method for rabbit control in pest control policy.

## Introduction

Invasive mammalian herbivores can cause significant agricultural and environmental damage and economic losses worldwide [1, 2]. One species, the European rabbit (*Oryctolagus cuniculus*), has been widely introduced to areas outside its native range [3–6] and has become a serious problem for agricultural production in New Zealand and Australia. In these countries rabbits have attained sufficiently high densities that they can damage soil and vegetation and degrade water quality [5–9]. They also impact native plants and animals through overgrazing, and altered predator–prey dynamics [10–14]. In order to supress rabbit infestations, land-owners or pest-control managers have developed and implemented various forms of population control; however, the typically high intrinsic rates of increase of rabbit populations can allow rapid recovery back to damaging levels, undermining control efforts. This creates an ongoing need for more efficient and affordable control methods.

In New Zealand the calicivirus that causes Rabbit Haemorrhagic Disease (RHD) was illegally introduced and released in 1997. In the two decades since then, the biological control afforded by RHD has been the major factor suppressing rabbit populations, but its effects are now waning in some places [7, 15, 16]. Thus, there has been a resurgence in the need for, and use of, lethal control methods [17], such as the example under consideration here—toxic bating. Toxic baits for rabbit control have traditionally been sown using bait-applicators towed behind a four-wheel motorbike [5, 18]. They have been employed to achieve complete bait coverage or to strip-sow bait in a series of strips ~20–40 m apart depending on the level of rabbit infestation [18]. However, these ground-based approaches are only feasible in areas with easy access and gentle terrain, and where rabbits are at low to moderate densities [5, 7, 17]. For vast areas of steep semi-arid mountain land, aerial baiting is the only feasible means of achieving effective control. In both New Zealand and Australia, aerial poisoning has been used since the 1950s to control rabbits [5, 7, 17, 19]. Aerial poisoning is often undertaken using diced fresh carrot baits coated with the toxin sodium fluoroacetate (‘1080’). Best Practice guidelines for aerial 1080 baiting for rabbits in New Zealand [18] currently stipulate the application of 20–40 kg of bait coated with 0.02% 1080 solution per hectare in areas with high rabbit infestations, and bait sowing so as to achieve complete and uniform coverage of the treated area.

This approach is highly effective at controlling rabbits over large areas [5, 16, 17], but it is expensive. This results from the high sowing rates of bait and the long flying times required to drop baits (costs of NZ$80–150 per ha are usual [7]; A.D.M. Latham, unpublished data). Moreover, the current high sowing rates also result in high application rates of active component on the ground, which in the case of 1080 raises concern among some sectors of the public around non-target impacts on indigenous wildlife, domestic livestock, pets, and recreational hunting species [20–23].

Here we aimed to determine whether sowing rates (and therefore costs) could be reduced by changing the way in which bait is distributed–specifically, by creating gaps in bait coverage (baiting in strips), in contrast to the current standard practice of achieving complete coverage. The underpinning hypothesis is that at the scale of a rabbit home range (typically a few hectares; [24]), bait density within baited strips is the primary determinant of kill success, rather than the average bait density over the whole area treated. Where the whole area is covered with bait, as is in standard practice, the two things are equivalent; but if bait coverage is only partial (but bait density within baited areas remains the same, or is even higher), less bait would be required overall because of the unbaited gaps. The rationale behind strip-sowing bait is similar to that behind the methods of laying baits in trails commonly used in Australia [5], but more specifically this hypothesis derives from recent research on two other major vertebrate pests in New Zealand that are also controlled using large-scale aerial 1080 baiting (brushtail possums *Trichosurus vulpecula* and ship rats *Rattus rattus*; [25]). Nugent et al. [26, 27] showed that there was little loss of possum control efficacy when 1080-bait was sown in widely spaced high-density strips or clusters, with unbaited gaps between them, provided these gaps remained smaller than the width of a possum home range. The key proviso is that bait density in the baited areas (bait foci) must be kept high–this is necessary because, due to unavoidable bait fragmentation during aerial deployment [28], it is possible that not all individual baits actually contain a lethal dose of 1080, therefore an animal that encounters and consumes a sub-lethal dose must then find and consume a second bait before the onset of toxicosis and consequent bait-aversion.

To address the issues of reducing the per hectare usage of 1080 [29], and the cost of rabbit control, we conducted a series of field trials to assess the efficacy of different aerial toxic-bait sowing methods. In describing these approaches, and in order to be consistent with the long-established terminology in New Zealand, we hereafter use the terms ‘broadcast’ and ‘strip-sowing’ as being synonymous with ‘complete coverage/uniform bait density’ and ‘partial coverage/concentrated bait foci’ respectively. To test our hypothesis, we looked at rabbit control using experimental aerial delivery of 1080-carrots in strip-sown patterns and compared this to best practice aerial broadcast sowing, in two rabbit-prone regions. We assessed overall efficacy of the operations in terms of reductions in rabbit counts, the per hectare cost of control and the quantity of toxin applied per hectare. We worked under the assumption that the efficacy of strip-sown bait depended on rabbit (winter) activity range [24] relative to the size of the unbaited gaps and the density of baits within the sown areas (i.e. the strips), and we took account of this in experimental design and data analysis. Finally, we modelled rabbit population recovery after control to estimate relative cost differences in strip- versus broadcast-sowing for agricultural production over a projected 20 year farm management plan.

## Methods

### Ethics statement

Our data collection complied with all relevant national laws of New Zealand. Experimental rabbit control operations trialled in this study were reviewed and approved by the Landcare Research Animal Ethics Committee (Code No. 11/04/03). Permission to conduct field work on private land was granted by the landowner for each of the study sites in this project.

### Overall design and study areas

**Fig 1:**
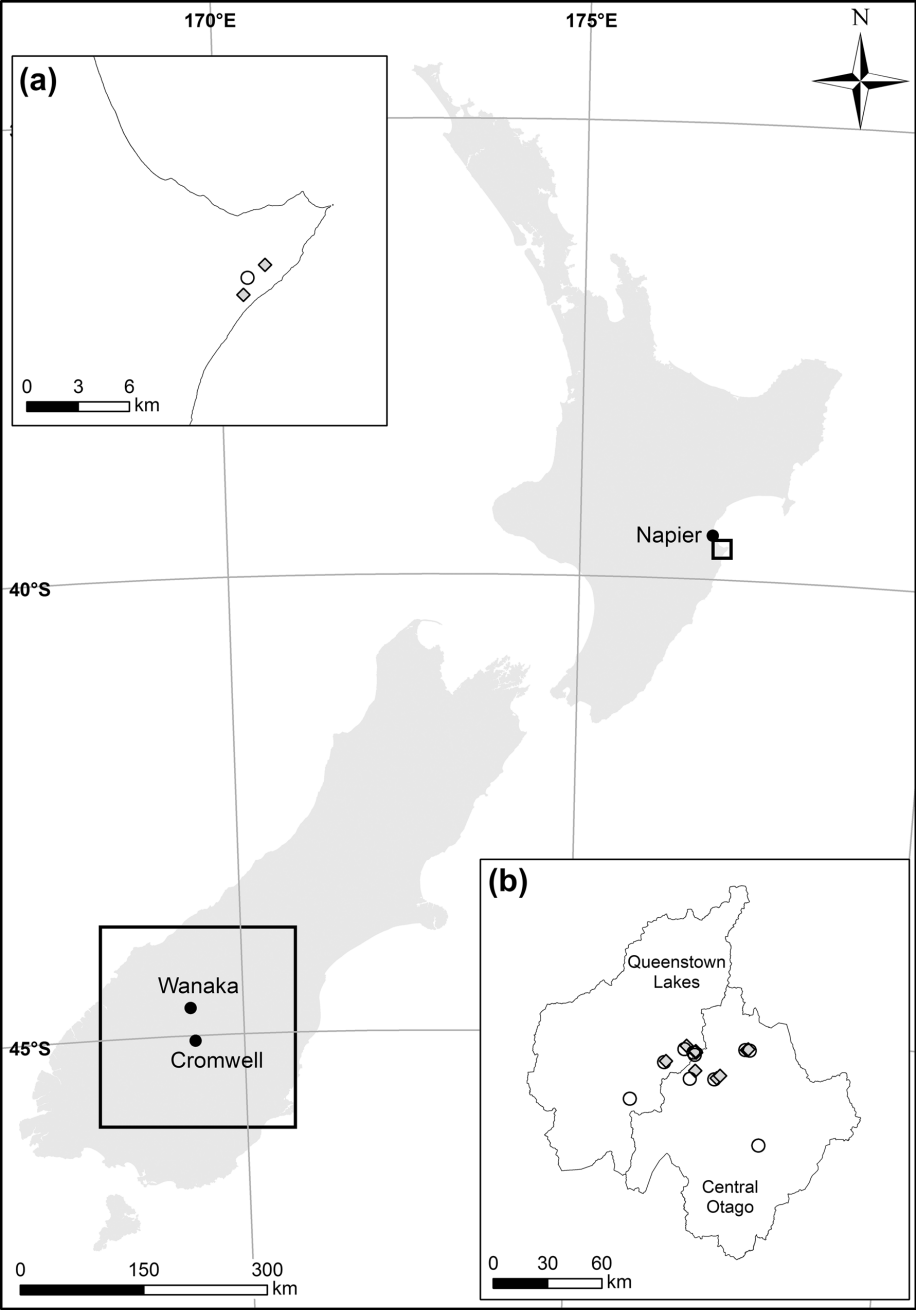
Location of the semi-arid areas in New Zealand where two methods of aerially-sowing 1080-bait to control rabbits were compared in trials in 2011–2014. In the North Island, 3 trials were conducted in 2013 within Cape Sanctuary near Napier in the Hawkes Bay region (a); in the South Island, 19 trials were conducted between 2011−2014 across a number of properties located in Central Otago and Queenstown Lakes districts (b). Location of trials using broadcast application of bait are shown as black circles, whereas those where strip-sowing was used are shown as grey diamonds.

We compared the outcomes of broadcast and strip-sowing treatments applied to twenty-two ~160 ha study blocks by counting rabbits (at night) along transects before and after aerial 1080 baiting, and then comparing the relative reduction in rabbit counts between the two treatments. The study blocks were located within two rabbit-prone regions: Central Otago and Queenstown Lakes districts, South Island (hereafter referred to as Central Otago); and Cape Sanctuary, Coastal Hawkes Bay District, North Island (hereafter referred to as Hawkes Bay), New Zealand (close to the town of Napier; Fig 1). Central Otago is semi-arid (mean annual rainfall 350−650 mm) and is characterized by cold winters (average mid-winter (Jul.) min. to max. = -1.5−8.0°C) and warm summers (average mid-summer (Jan.) min. to max. = 11.0−24.4°C). The region comprises short-tussock grassland interspersed by shrubs and pastureland [8] and supports high rabbit populations that generally exceed a spotlight count index of 40 rabbits per linear km [7, 30, 31]. Hawkes Bay is also relatively dry land (mean annual rainfall 784 mm) and has mild winters (average mid-winter min. to max. = 5.6−14.1°C) and warm summers (average mid-summer min. to max. = 14.6−24.5°C). Our study sites in this region were on agricultural land with surrounding patches of native forest in the Cape Sanctuary wildlife sanctuary [32], habitat that supports medium to high rabbit numbers (i.e. spotlight count indices generally of 10–20 rabbits per linear km [7, 30]).

### Pre-trial rabbit populations

Our studies were conducted in conjunction with (and under guidance from) local regional councils, whose field workers provided up-to-date population monitoring information. This included an *a priori* indication that epidemics of viral RHD had occurred in both regions after 1997, however by the start of our studies local rabbit populations had developed sufficient resistance to have returned to pre-RHD levels [7, 15, 16]. Immediately prior to trial commencement, rabbit population densities at the Central Otago sites were estimated to be at the high level of 5–6 on the modified McLean Scale field measure of rabbit density [33], and at the Hawkes Bay sites to be at the very high level of ≥ 7 on this scale [18].

### Trial design and control procedures

Start and end dates for rabbit control depended on temperatures sufficiently cold to stop plant growth and encourage rabbit feeding on carrot bait [34], typically between late-June and mid-September. Central Otago trials were undertaken on 11 different sites in the winters of 2011–2014, and Hawkes Bay trials on 2 sites in winter 2013. We conducted ten broadcast replicates (mean block size = 155.3 ha, range: 101.7−244.8) and nine strip-sown replicates (mean = 158.3 ha; range: 100−454.7) in Central Otago. Where possible treatments were paired in the same site, however in some sites more than one replicate of a given treatment had to be conducted. We conducted one broadcast replicate (103 ha) and two strip-sown replicates (mean = 226 ha) in Hawkes Bay (Fig 1). Aerial baiting followed current Best Practice to encourage bait-familiarization (‘pre-feeding’ [35]): this comprised two baiting sessions of unpoisoned carrot 5–7 days apart [18], with full details of delivery, dosage and sowing pattern shown in Table 1.

**Table 1:** Specifications of the two treatments assessed for the aerial control of rabbits in two semi-arid regions (Central Otago and Hawkes Bay) in New Zealand.

Treatment	Pre-feed 1	Pre-feed 2	Toxic sowing rate (kg/ha)^1^	Intended strip width (m)	Flight path spacing (m)
Strip-sown	Broadcast	Strip-sown	10 (15)	10	75
Broadcast	Broadcast	Broadcast	30 (35)	25	25

Pre-feeds were of non-toxic carrots and were intended to familiarise rabbits with carrot as a bait. The first pre-feed for both treatments was broadcast at 30 kg per hectare (35 kg/ha in Hawkes Bay), whereas the second strip-sown pre-feed was 10 kg per hectare (15 kg/ha in Hawkes Bay). The flight path spacing indicates how far apart the flight lines of a fixed-wing aircraft need to be to achieve complete coverage of a treated area (broadcast) and a strip-sown treatment with an intended strip of 10 m and an unbaited gap of 65 m.

^1^ Values shown in parentheses are for Hawkes Bay

**Fig 2:**
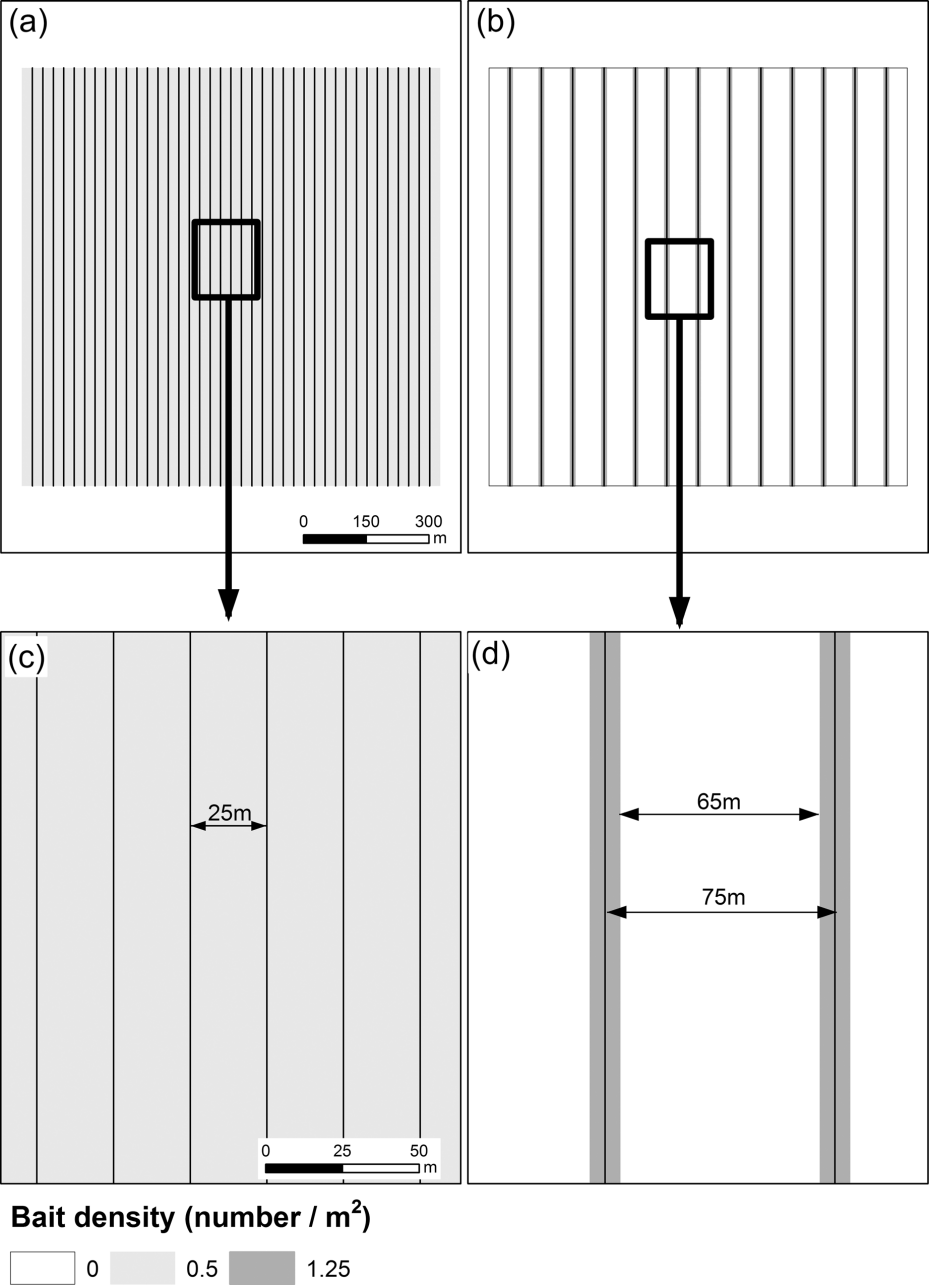
Schematic diagram of two methods of aerially-sowing 1080-covered carrot baits to control rabbits within 100 ha experimental plots in semi-arid regions in New Zealand. Broadcast baiting (a) entails complete coverage of the area with carrot baits at a density of 0.5/m^2^. Using a fixed-wing aircraft, this pattern was achieved by deploying strips of baits every 25 m (i.e. the flight path spacing; FPS), with each delivery of bait having a swath width of 25 m (c). This treatment leaves no unbaited gaps. Strip-sowing (b) entails partial coverage of the area with carrot baits at a target density of 1.25/m^2^ within the baited strips. This was achieved by deploying strips of baits every 75 m (i.e. the FPS), with each strip having a target swath width of 10 m (d). This treatment leaves unbaited gaps of 65 m in width between the outer extremities of swaths of bait.

To produce bait, a Reliance bait cutter fitted with a 29 mm grill [36] and a 19 mm drum screener were used to produce average 6 g dices of carrot. Toxin-loading was achieved by coating carrots with 1080 (aqueous form) to a final concentration of 0.02% wt/wt 1080:carrot [17]. Toxic bait was sown 5–7 days following the second pre-feed. In Central Otago, 1080-carrot bait was broadcast-sown at 30 kg/ha as per recommended best practice for rabbit populations at the modified McLean Scale of 5–6 (Table 1; [18]). For strip-sowing in the same area, 1080-carrot bait was applied at 10 kg/ha by using GPS-guidance to deploy bait in strips aiming to be 10 m-wide with a flight path spacing (FPS) of 75 m between the strips (Table 1, Fig 2). In Hawkes Bay, the same procedures were followed but with elevated sowing rates to 35 kg/ha and 15 kg/ha for broadcast- and strip-sowing, respectively, to account for the higher estimated rabbit densities. We used a fixed-wing aircraft (Walter turbine-powered Fletcher) to sow bait in 11 treatment blocks in Central Otago. Where there was no nearby airstrip or other restrictions applied, we instead used a Bell 206 helicopter fitted with either of two buckets, namely one with a conventional bait spinner for broadcasting [27] or one set up to trickle-feed bait for strip sowing in ~10 m swaths. Although the mechanisms by which baits were sown from a helicopter differed slightly, they achieved similar patterns of bait for broadcast and strip-sown treatments as a fixed-wing aircraft (Table 1): in total, 8 blocks in Central Otago were sown via helicopter and 3 in Hawkes Bay. Prior to trial commencement, practice-runs were performed over flat fields and the distribution pattern and size of bait deployed by each aircraft type was determined by hand-collecting baits from the ground.

### Field data acquisition and analysis

Within each treatment block we established four monitoring transects of 800 m in length, each at least 200 m from the next nearest transect and 100 m from the edge of the block. This was done to avoid double-counting rabbits on transects and to reduce edge-effects. We conducted spotlight transect counts starting 30 min after sunset [16, 37]. Transects were walked by a single observer who counted the number of rabbits seen over ~70 m (depending on terrain) either side of a 180° arc created using a 50- or 75-W hand-held spotlight. Spotlight transects were surveyed on two consecutive nights pre- (c. 1 week before the control operation) and post-control (c. 2 weeks after) by the same observer using the same equipment and, as far as possible, in the same weather conditions to minimise non-treatment biases.

We recorded rabbit numbers in the Hawkes Bay trials but excluded these data from any formal statistical analyses because of the higher sowing rates used at this location and the low level of (or lack of) replication (see Results). For Central Otago, the numbers of rabbits seen per 800m transect were averaged across the two surveyed nights, and between the four transects to obtain one estimate of rabbits pre- and post-treatment for each replicate. For efficacy comparisons, the mean percentage kill was compared between strip-sown and broadcasting methods using a t-test. An ANCOVA was then used to assess whether the mean number of post-control survivors per replicate was related to the initial number of rabbits recorded, the treatment (strip vs. broadcast sowing), and whether there was an interaction between these two factors; i.e., to assess whether there was any evidence that the amount of bait sown under each treatment had been insufficient to kill all of the rabbits present. We log-transformed both continuous variables (pre- and post-treatment rabbit counts) prior to this analysis.

Finally, to assess whether environmental factors (as opposed to experimental treatments) had affected the percentage kill attained at each spotlight transect, we used a linear mixed-effects regression (using the function lmer in package lme4 in R [38]). The environmental factors included in this analysis were: type of aircraft used (fixed-wing or helicopter), average distance to nearest edge of unpoisoned land, initial relative abundance of rabbits, treatment, terrain ruggedness (estimated as the standard deviation of elevation along the transect), and proportion of the transect traversing scrub habitat. Terrain ruggedness was derived from a 25 m resolution digital elevation model of the study area. The length of transect traversing habitat covered by scrub was extracted from the New Zealand Land Cover Data Base (LCDB version 4; www.lcdb.scinfo.org.nz) by identifying scrub as polygons belonging to the following land cover classes: shrublands, matagouri (*Discaria toumatou*) scrub, and mānuka/kānuka (*Leptospermum scoparium*/*Kunzea ericoides*) stands. Prior to analyses, we logit-transformed (log(*y* / [1 –*y*])) the dependent variable (percent reduction in rabbit counts) to fulfil linear modelling assumptions and added the minimum non-zero value for (1 –*y*) to sample proportions equal to 1 (which would otherwise transform to ∞ [39]). We used each transect (n = 76) as a replicate and block as the random effect in regression analyses.

### Rabbit population recovery modelling

In practical terms, the long-term efficacy or cost-effectiveness of aerial 1080 baiting is determined by how quickly the residual population recovers to the level at which repeat control will be required. To assess the likely recovery of rabbit abundance after control, we constructed a population simulation model following Barlow and Kean’s model 2.3 [40]. Although rabbit populations in New Zealand sometimes experience large and sudden reductions due to epidemics of RHD [41], we did not consider any RHD effects directly in our model because (1) there is evidence of current widespread immunity to the disease in rabbits in Central Otago [15], and (2) even if an outbreak of the disease did occur, it should affect rabbit populations controlled under a strip-sown or broadcasting scenario equally. Instead we indirectly accounted for possible residual effects of RHD in our study area by modelling rabbit dynamics using two alternative values for the instantaneous rate of increase (r): (1) using the value from Barlow and Kean [40] for a pre-RHD population (r_pre-RHD_), and (2) half that value for a post-RHD population (r_post-RHD_). This r_post-RHD_ was chosen to reflect the lower population growth rates following RHD epidemics reported by Parkes et al. [15]. The remaining population parameter values used in the model were from Table 1 of Barlow and Kean [40] for New Zealand semi-arid environments.

In the model, rabbit control is applied on 1 July of the first year of the simulation, and the efficacy of the control operation (percentage kill) is drawn from a beta distribution. The two parameters for the beta distribution were determined from the mean percentage kill and variance obtained for the strip-sown and broadcast treatments separately. The model was run under four scenarios: pre- and post- an RHD epidemic, and strip and broadcast sowing treatments. The model was run 10,000 times for each scenario, and for each simulation, we calculated the number of years until the rabbit population that underwent control reached pre-control densities. The mean number of years obtained under each scenario was then converted into estimated relative costs (per hectare per year) over a 20 year farm management plan. To do this, we assumed that the cost of control per hectare for the broadcast treatment was $NZ 100 and that the strip-sown treatment cost 25% less ($NZ 75) than broadcast, based on two thirds less bait and lower fixed-wing flight time.

All analyses and simulations were undertaken in the R Statistical Computing Environment, version 3.1.3 [42].

### Data accessibility

Data presented in this study are publicly accessible at http://datastore.landcareresearch.co.nz/dataset/raw-data-for-manuscript-by-latham-et-al-refining-operational-practice. The data sets refer to 1] pre- and post-control numbers of rabbits from 22 replicate study runs and percentage kill estimations from these, and 2] modelling simulation data generated from 10,000 iterations of pre- and post- control (with and without the co-impact of RHD on the rabbit populations).

## Results

### Swath width and bait size distribution

During practice-runs using the two types of aircraft, the effective baited swath width (excluding 5% of outlying baits) achieved using a fixed-wing aircraft over a flat field was approximately 24 m (12 m either side of the flight line), while the effective swath width achieved using a helicopter over a flat field was approximately 13 m. Although the swath width for the strip-sown treatment was wider than planned, the recorded bait density (1.13 baits per m^2^) in the strip areas represented concentrated bait foci at a higher density than that estimated for broadcasting (0.5 baits per m^2^ over the entire treated area). Of 1,931 carrot baits that were collected and weighed, 46% were < 4 g, 36% were 4–6 g, and 18% were > 6 g.

### Reductions in rabbit counts

**Fig 3:**
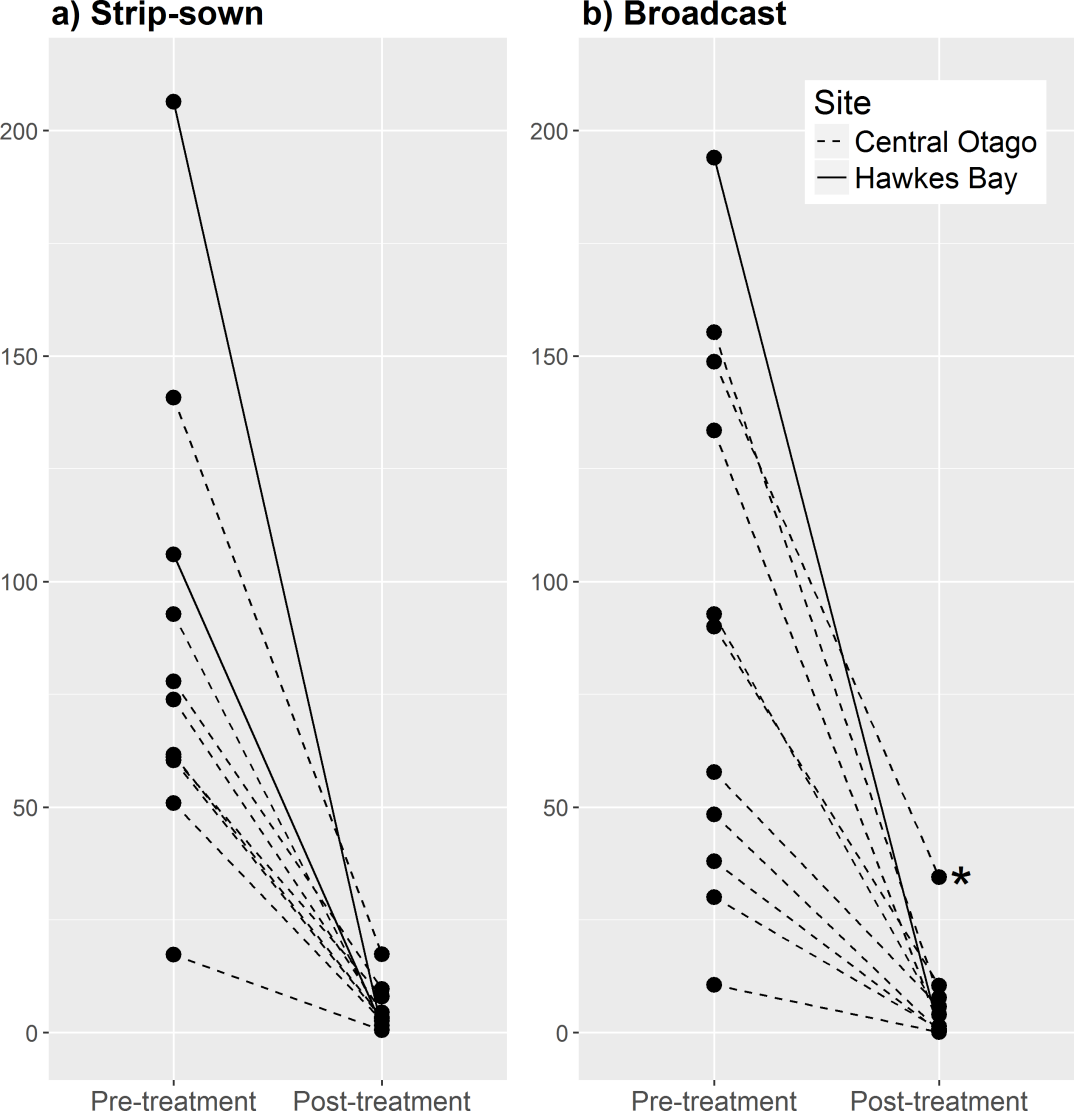
Relative reductions in rabbit numbers, as assessed by pre- vs post-treatment changes in the number of rabbits seen on 800 m transects, following strip-sown or broadcast application of 1080-bait in large blocks in Otago (n = 19) and Hawkes Bay (n = 3) between 2011 and 2014. The lines represent the decline in rabbit activity between pre- and post-treatment periods, the asterisk demarks the single broadcast operation (in 2012) when an anomalously low kill rate of 77% was achieved.

The highest number of rabbits counted on a single 800 m transect was 269 and was recorded in Central Otago before control. Pre-control rabbit counts were higher in Hawkes Bay (mean = 170 rabbits/transect; Fig 3A and 3B) than in Central Otago (mean = 76 rabbits/transect; Fig 3A and 3B).

The estimated percentage kill attained on Hawkes Bay was very high for both strip-sown (98.9%) and broadcast (99.8%) treatments. Because higher sowing rates were used in this area than in Central Otago, these data were not included in any of the statistical analyses described below.

In Central Otago, the average number of rabbits seen per 800 m transect before control ranged between 17.2 and 140.8 in the strip-sown treatment blocks, and between 10.6 and 155.2 in the broadcast treatment blocks (Fig 3A and 3B). The number of rabbits seen per 800 m transect after control ranged from 0.5 to 17.4 for the strip-sown treatment blocks, and from 0.1 to 34.5 in the broadcast treatment blocks (Fig 3A and 3B). The average difference in the number of rabbits seen on the same transect over the two consecutive nights of monitoring was 18 before control and 2 after control.

The overall mean reduction in rabbit numbers achieved by aerial 1080 baiting in Central Otago was 93.4% with all but one replicate exceeding 87% (an anomalously low 77% kill was recorded from a single broadcast replicate). The mean reduction in rabbit numbers for the strip-sown treatment blocks (92.7%; 95% CI = 90.0; 95.5) and the broadcast treatment blocks (94.0%; 95% CI = 89.5; 98.4) did not differ significantly (t_14.71_ = 0.47, P = 0.65). A power analysis on the observed 1.3% point difference in kill rate between the two treatments suggests that a minimum of 350 replicate trials would be required in Central Otago to determine with reasonable power (80%) whether the difference between the two treatments actually exists; cost estimates for such a study exceed $NZ40 million.

**Fig 4:**
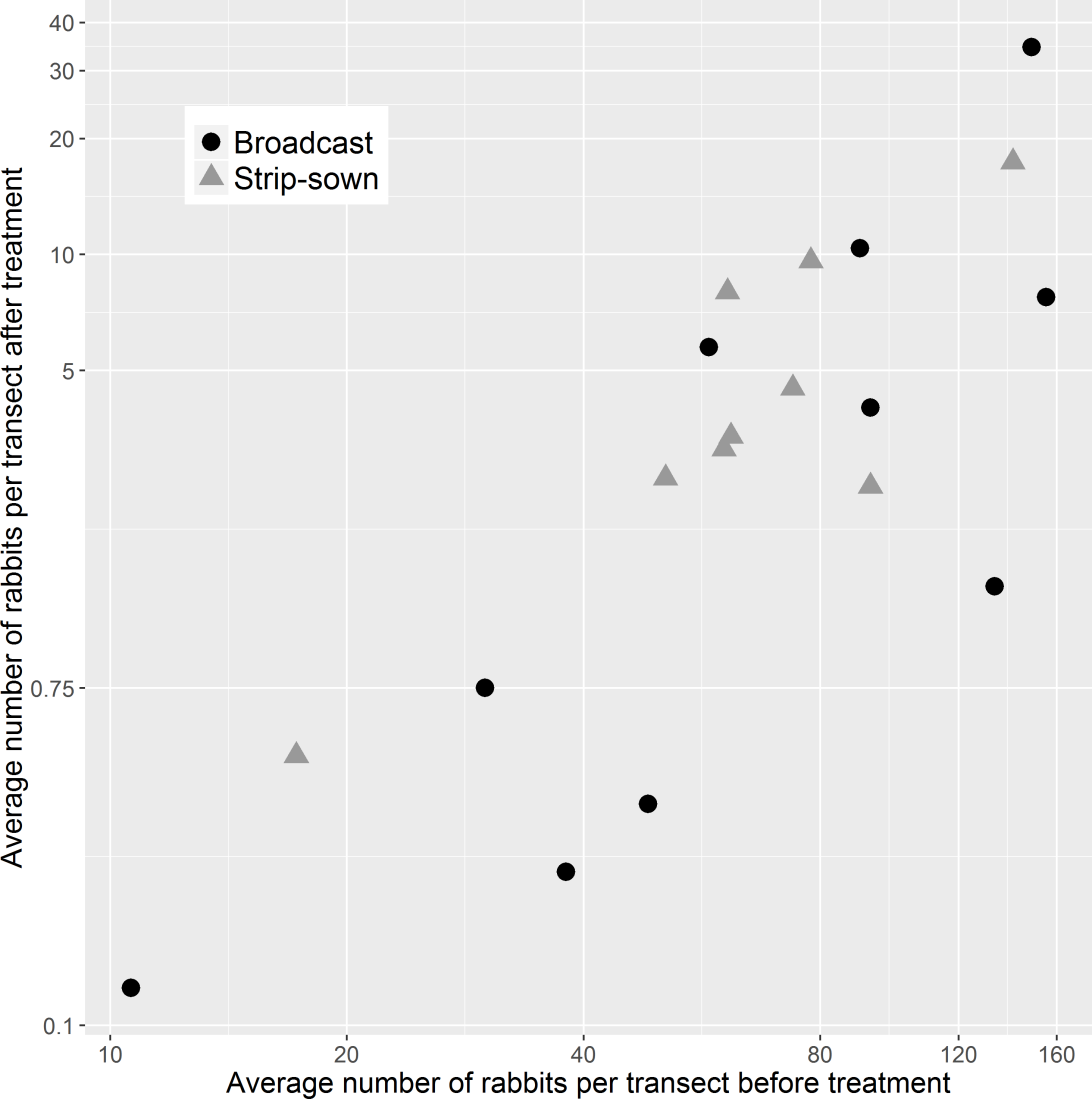
Number of rabbits recorded following aerial 1080-bait treatment as a function of the initial number of rabbits recorded on 800 m transects, in Central Otago and Queenstown Lakes districts, New Zealand, 2011–2014. Studies were conducted over 19 large blocks, with the application of 1080-bait done using either strip-sowing or broadcasting. The x-axis and the y-axis are in log-scale.

The number of post-control surviving rabbits was not significantly different between treatments (F_1, 15_ = 2.66, P = 0.12), but was significantly higher at sites with higher initial rabbit counts (F_1, 15_ = 31.23, P < 0.01; Fig 4), indicating that the poorest reductions in rabbit counts were recorded at sites with the highest pre-control counts. However, the interaction between these two factors was not significant (F_1, 15_ = 0.11, P = 0.75), i.e., there was no indication of a stronger positive relationship between pre- and post-treatment rabbit counts under the lower overall sowing rates used for the strip-sowing treatment (Fig 4). The proportion of rabbits killed on each transect was not significantly affected by any of the environmental factors that we assessed (based on α = 0.05, Table 2); however, terrain ruggedness had a significant negative effect based on α = 0.1.

**Table 2:** Parameters for fixed-effects included in a linear mixed-effect model, used to assess the influence of landscape variables and 1080-bait aerial sowing method on the proportion of rabbits killed at each 800 m transect surveyed (N = 76) in Central Otago and Queenstown Lakes districts, New Zealand, 2011−2014.

Variable	β	S.E.	t-value	P
Intercept	1.44122	0.23709	6.07900	<0.001
Treatment^1^	-0.28640	0.18038	-1.58800	0.13960
Rabbits before^2^	-0.00053	0.00132	-0.40400	0.68780
Av. distance^3^	0.00016	0.00031	0.51600	0.60780
Terrain ruggedness^4^	-0.00648	0.00357	-1.81700	0.07360
Aircraft^5^	0.29591	0.18687	1.58300	0.13900
Closed habitat^6^	0.72465	0.55529	1.30500	0.19620

The model included a random effect for block (i.e., there were four transects surveyed within each block). The dependent variable was logit-transformed (log(*y* / [1 –*y*])) prior to analyses in order to meet the assumption of normality.

^1^ Categorical variable indicating whether the application of 1080 was done by broadcasting (reference category) or strip-sown.

^2^ Number of rabbits per 800 m transect recorded before the treatment.

^3^ Average distance to nearest edge of unpoisoned land (m).

^4^ The standard deviation of elevation along transects.

^5^ Categorical variable indicating whether the application of 1080 was done using a helicopter (reference category) or a fixed-wing aircraft.

^6^ Proportion of the transect traversing habitat covered by scrub (including shrubland, matagouri scrub and mānuka stands)

### Simulations of rabbit population recovery

For a rabbit population with a pre-RHD rate of increase, simulations suggested control would have to occur every 5.5 years using a strip-sown treatment or 6.3 years using broadcast baiting (Table 3). Over a 20-year farm management plan, this would translate into costs of $NZ 13.58 and $NZ 15.91 per hectare per year, respectively, for the two sowing methods. For rabbits to be controlled on a 2,000 hectare farm in a pre-RHD environment, a strip-sown application of 1080 would cost $NZ 4,650 less per year, and $NZ 93,000 less over a 20-year farm plan, compared to broadcast baiting. Similarly, for a rabbit population with a post-RHD rate of increase, control would have to occur every 9.6 years using a strip-sown treatment or 11.1 years using broadcasting (Table 3); this translates to costs of $NZ 7.82 and $NZ 8.99 per hectare per year, respectively, for the two sowing methods. Over a 20-year farm plan for a 2,000 ha farm in a post-RHD environment, simulations predict that strip-sowing of 1080 would save $NZ 46,660 in pest-control operational costs compared to broadcast baiting.

**Table 3:** Cost (per ha per year) of 1080-bait control of rabbits using two alternative aerial-delivery methods over a 20-year farm management plan in Central Otago and Queenstown Lakes districts, New Zealand.

Population rate of increase (r)	1080 Treatment	Frequency of 1080 operations (years)	Cost per ha per operation ($NZ)	Number of 1080 operations over 20 years	Cost per ha over 20 years ($NZ)	Cost per ha per year ($NZ)
Pre-RHD	Strip-sown	5.5	75	3.62	271.64	13.6
	Broadcast	6.3	100	3.18	318.14	15.9
Post-RHD	Strip-sown	9.6	75	2.09	156.43	7.8
	Broadcast	11.1	100	1.80	179.75	9.0

Costs were calculated based on the frequency with which 1080 operations would have to occur, which was derived from a model of rabbit population growth, using two alternative rates of increase (pre-RHD and post-RHD) and parameters for the efficacy of the control operation based on the empirical values obtained from 9 strip-sown and 10 broadcasting trials conducted between 2011−2014. Calculations assume that the cost per hectare of the strip-sown treatment is 25% less than the broadcasting treatment.

## Discussion

Our primary objective in this study was to compare the operational efficacy of two different aerial sowing techniques of 1080-bait for controlling rabbits in high population settings: either broadcast sowing (which is current best practice) or strip-sowing (which has been shown to be a cost-effective alternative against another invasive mammalian pest, the brushtail possum [26, 27]). Both sowing methods proved highly effective and we found no difference in the percentage of rabbits killed using either treatment. The small difference in efficacy we recorded between the two methods (1.3 percentage points) is unlikely to be of significance to practical operations in the short term. However, if we hypothetically apply this difference in 20 year simulations of post-control rabbit population increases, it is apparent that one effect would be marginally quicker rabbit population recovery to pre-control numbers under strip-sowing compared to broadcast-sowing (i.e. about 1–1.5 years quicker over a 6 or 11 year period for pre- or post-RHD simulations, respectively). However, in economic terms, the slightly greater frequency of repeat control required under strip-sowing would be offset by the lower cost of this approach—we predict savings of approximately $NZ 50,000–100,000 (depending on the state of RHD epidemics) over a 20-year farm management plan, assuming a conservative operational cost-saving per ha of 25% under strip-sowing.

It is important to emphasise that the above cost projections did not take into account costs for secondary follow-up control of survivors used by some farmers to slow population recovery and prevent ongoing damage by rabbits surviving aerial baiting. This would likely have reduced the small difference in percentage kill between treatments even further. Had we considered this in our estimates, the time intervals between repeat control would be greatly extended for both methods and the per hectare per year savings accrued through using strip-sowing could have been even greater than those estimated here.

None of the environmental variables that we assessed had a significant effect on the efficacy of rabbit control. However, we did notice that residual rabbits observed after aerial 1080 baiting were often in close proximity to unpoisoned edges. It is unclear whether these were resident rabbits that survived the operations, or animals that had moved into the control area from adjacent habitat. Further, our analysis provided some support that the percent kill achieved was lower in transects with more rugged terrain. From a practitioner’s viewpoint, we would recommend caution in applying strip-sowing control in circumstances where it may be difficult to align strips sufficiently to ensure all rabbits are able to encounter sufficient bait (i.e. to ensure that there are no gaps between baited areas of more than ~50 m). This might be of particular concern in areas that are comparatively small (<100 ha), irregularly shaped, and/or where the terrain is broken and rugged. The full exposure of all animals to a sufficient quantity of bait to ensure lethality within a short time frame has been identified previously by Nugent et al. [17] as a key factor in determining successful aerial baiting strategies.

An important benefit (in addition to the reduction in cost) of strip-sowing is the substantial reduction in the amount of toxin being used per hectare (~two thirds less 1080 using a FPS of 75 m and a sowing rate of 10 kg/ha). That could have important benefits for native biodiversity and agriculture [23]. First, it directly addresses a key recommendation of the 2011 review of 1080 use in New Zealand: to optimise the use of 1080 to minimise potential risks associated with the toxin [29], in particular a reduced risk of primary poisoning of non-target native wildlife (e.g. kea *Nestor notabilis*, paradise shelduck *Tadorna variegata*, and possibly reptiles and invertebrates) and recreational hunting species (e.g. fallow deer *Dama dama*, chukar *Alectoris chukor*, and California quail *Callipepla californica*) that can occur in rabbit-prone areas [23, 43]. Given the lower bait application rate used under strip-sowing, coupled with the knowledge that rabbits can consume tens of carrot baits in rapid succession if presented with the opportunity [17], it is reasonable to assume that they would eat a large proportion of the baits deployed with this sowing method. This should result in fewer residual baits and thus a reduced likelihood of non-target poisoning compared to broadcast sowing [23]. Hence secondly, fewer residual toxic baits may also permit shortening of the mandatory stand-down period of 10 weeks (or 100 mm of rain) before landowners can return stock to 1080-treated land [44]. Reducing 1080 usage and baiting densities should address some of the concerns of those opposed to aerial use of this toxin [45]. The ongoing availability of 1080 is important because, in the post-RHD environment in New Zealand where population resistance to the disease is high [15], there are currently few other high-efficacy broad-scale tools for reducing high density rabbit populations [5, 7, 17].

Although our study was designed primarily to investigate the efficacy of two different aerial sowing patterns of toxic bait for rabbit control, we also made some relevant observations regarding the mode by which baits are delivered. In particular, strips sown by fixed-wing aircraft were observed to be almost twice as wide as those done by helicopter. This difference likely occurred because fixed-wing aircraft, particularly if not fitted with fins or wing fences, are affected by spanwise flow along the wing which creates vortices that, in our case, served to increase strip width or at least resulted in an inability to reduce strip width (J. Bishop, Otago Airspread Ltd, pers. comm.). We surmise that percentage kill was not affected by increased strip width because the recorded bait density in the concentrated bait foci of the treated area remained comparatively high (1.13 baits per m^2^), and thus rabbit:bait encounter rates were unlikely to have been reduced to a level where sub-lethal toxicosis would initiate a stop-feeding response before the ingestion of a lethal dose [46]. If all baits had been exactly 6 g in weight/size, and the deployment strips had been 10 m wide (as intended at outset; Table 1), the effective bait density would have been 1.25 per m^2^, but this is only marginally higher than that which we recorded in an actual average of a 23 m strip (1.13 baits per m^2^). An aircraft’s height above ground is likely to be highly variable in rugged terrain and this may influence the effect that spanwise flow and perhaps helicopter downwash have on the lateral flow of bait and thus strip width and bait density. For example, Nugent and Morriss [27] found that flying speed, height above ground, and topography resulted in differences in the mean length and width of clusters of cereal bait sown from a modified-bucket slung under a helicopter. Although the on-the-ground pattern of bait might differ because of these factors, such effects are unlikely to have been sufficiently large to reduce rabbit encounter rates with bait to the point where they could not find sufficient bait to obtain a lethal dose before the onset of toxicosis.

We found that the number of rabbits surviving was significantly related to the initial rabbit relative density (i.e. the pre-control rabbit counts), and it is interesting to note that the lowest percentage kill was recorded in the broadcast-sown block that had the highest pre-control counts. However, there was no indication in our data that this was directly related to baiting density (i.e. the number of baits available per rabbit), which we estimate to be at least in the hundreds (see above and [17]). This result, in conjunction with the fact that both strip- and broadcast-sowing methods had comparable control efficacy, is therefore consistent with our conclusion from prior 1080-control work on brushtail possums [26, 27] that while high local bait density is required to achieve a high percentage kill, it is not required over the whole treated area. Nevertheless, further assessment of the relationship between rabbit abundance and control efficacy is recommended, to determine at what relative abundance of rabbits a given application is too low to achieve a consistently high percentage kill (such as the 90–95% commonly targeted by pest-control operators). The New Zealand National Pest Control Agencies best-practice guidelines solution to this is to use a sliding-scale sowing rate to account for variable rabbit infestations [18], i.e. 10–40 kg of 1080-bait per hectare broadcast baiting for medium to very high infestations, respectively. We increased the toxic sowing rate for the strip-sown treatment at Hawkes Bay from 10 to 15 kg per hectare to account for a very high rabbit infestation at that site, and achieved very high kill rates (although because we did not have a lower sowing rate for comparison, we cannot be certain whether the increased rate was necessary in this case). Nevertheless, we recommend that a sliding scale for bait sowing rate should be considered for strip-sowing 1080-bait for rabbits, while noting that further data from control of high density populations are needed to verify this requirement and to determine appropriate sowing rates; this could be done by adopting an adaptive management approach. A sliding scale could also include sowing rates lower than the 10 kg per hectare used here, especially where rabbits at low densities are controlled to protect native plants that are susceptible to even low- to moderate-browsing [47].

Refining operational practices for controlling rabbits on agricultural lands can decrease both the cost of control and toxin use on a per unit area basis. We found that moderate to high savings could be accrued from using strip- rather than broadcast-sowing for rabbit control over a 20-year farm plan. We suggest that strip-sowing should be considered as an additional Best Practice method for controlling rabbits in New Zealand, and perhaps Australia, where the use of aerially-applied 1080 in bait is also permissible to control introduced rabbits. More broadly, the adoption of strip-sowing methodology (and its ensuant reduction in toxin use in the environment) may also have implications for mammalian pest control using aerial sowing in other circumstances: at least in an experimental setting it has been used to reduce toxin deployment rates for control of ship rats and brushtail possums in New Zealand [25–27], and could feasibly be used in an international setting, e.g. to reduce rates of aerial bait-delivered anticoagulants for invasive rodent control on conservation islands where bait is currently broadcast-sown [48, 49].
